# The efficacy and safety of mirabegron and α-adrenergic receptor antagonist in the treatment of distal ureteral stones: a systematic review and meta-analysis

**DOI:** 10.3389/fphar.2025.1517979

**Published:** 2025-03-19

**Authors:** Yicheng Guo, Fengze Sun, Yini Wang, Yanfei Li, Tianqi Wang, Xiaohong Ma, Jitao Wu

**Affiliations:** ^1^ Department of Urology, Yantai Yuhuangding Hospital, Qingdao University, Yantai, Shandong, China; ^2^ The Second Clinical Medical College, Binzhou Medical University, Yantai, China

**Keywords:** mirabegron, ureteral stone, α-adrenergic receptor antagonist, meta-analysis, β3adrenergic receptor mirabegron, β3adrenergic receptor

## Abstract

**Introduction:**

To systematically evaluate the efficacy and safety of mirabegron compared to α-adrenergic receptor antagonists for treating distal ureteral stones.

**Methods:**

A comprehensive search of EMBASE, PubMed, and Cochrane databases was conducted to identify studies comparing mirabegron and α-adrenergic receptor antagonists for stone expulsion. The primary outcome was stone expulsion rate (SER), and secondary outcomes included stone expulsion interval (SEI) and pain episode frequency. Risk ratio (RR) and mean differences (MD) with 95% CIs were calculated.

**Results:**

Six studies involving 487 participants were included. There was no significant difference in SER between mirabegron and α-adrenergic receptor antagonists (RR = 1.06; 95% CI = 0.93–1.22; P = 0.34). SEI showed no significant difference either (MD = 0.05; 95% CI = −3.23 to 3.34; P = 0.58). However, pain episodes were significantly reduced in the mirabegron group (MD = −0.36; 95% CI = −0.63 to −0.09; P = 0.01). Subgroup analysis showed reduced pain episodes with mirabegron versus silodosin but not tamsulosin. Mirabegron also had fewer side effects like headache (RR = 0.34; 95% CI = 0.13–0.87; P = 0.02) and orthostatic hypotension (RR = 0.11; 95% CI = 0.02–0.55; P = 0.008), while dizziness and ejaculation dysfunction rates were comparable.

**Conclusion:**

Mirabegron reduced pain episodes during treatment for distal ureteral stones, particularly when compared to silodosin, despite no significant differences in SER or SEI. Its favorable safety profile suggests potential as a therapeutic option. Further randomized controlled trials are needed to confirm these findings.

## Introduction

Urolithiasis is a common condition affecting the human urinary system, with a prevalence of approximately 2%–3% across all populations (Cui et al., 2014). Ureteral stones, which make up about 20% of all urolithiasis cases, are particularly troublesome, with around 70% of these stones located in the distal ureter (Ahmed and Al-Sayed, 2010; Raheem et al., 2017). If untreated, ureteral stones can cause severe pain and lead to serious complications, such as acute kidney injury, infections, and septic shock, posing significant health risks and economic burdens for patients (Thongprayoon et al., 2020; Türk et al., 2016).

The main treatment options for ureteral stones include medical expulsion therapy (MET), extracorporeal shockwave lithotripsy (ESWL), and endoscopic surgery (Paffenholz and Heidenreich, 2021). MET is commonly recommended for distal ureteral stones, as it helps relax smooth muscles in the urinary tract, facilitating the passage of stones (Segura et al., 1997). The most widely used medications for MET are α-adrenergic antagonist, such as tamsulosin (Raheem et al., 2017).

Recently, mirabegron, a β3-adrenergic receptor agonist, has gained attention for its ability to relax bladder smooth muscle and alleviate overactive bladder symptoms (Solakhan et al., 2019; Wanajo et al., 2004). Emerging research indicates that β3-adrenergic receptors are also expressed in the smooth muscle and urothelium of the ureter, suggesting that mirabegron could be a novel option for MET in treating ureteral stones (Kaya et al., 2018; Kelleher et al., 2018). Activation of β3-adrenergic receptors can reduce the excitability of smooth muscle cells by inhibiting the release of intracellular calcium ions (Ca^2+^) and enhancing the efflux of potassium ions (K^+^), which may contribute to the relaxation of ureteral smooth muscle and facilitate stone passage (Dey et al., 2024). However, the current evidence regarding its effectiveness remains inconclusive and has yet to be thoroughly evaluated. This study aims to systematically review and compare the effectiveness and safety of mirabegron versus α-adrenergic receptor antagonist in the treatment of distal ureteral stones.

## Methods

The review protocol was registered with PROSPERO (CRD42024599866; https://www.crd.york.ac.uk/PROSPERO/) following the guidelines outlined in the Preferred Reporting Items for Systematic Reviews and Meta-Analyses (PRISMA).

### Search strategy

We performed a comprehensive literature search in the PubMed, Embase, and Cochrane databases, adhering to the PRISMA (Preferred Reporting Items for Systematic Reviews and Meta-Analyses) guidelines (Page et al., 2021) (Supplementary Table 1). The search covered publications from the databases’ inception to August 2024. Our retrieval strategy was formulated using the PICOS framework (population, intervention, comparators, outcomes, and study design). The key terms used in the search included mirabegron, β3-adrenergic receptor agonists, tamsulosin, silodosin, α-adrenergic receptor antagonist, and ureteral stones. We limited the search to English-language articles and randomized controlled trials (RCTs). Two authors independently conducted the searches following the established strategy, and their results were cross-checked. All identified articles were evaluated separately by two reviewers, with any disagreements resolved by consulting a third researcher. Furthermore, relevant references from the included studies were also reviewed where necessary.

### Inclusion criteria and data extraction

The inclusion criteria for all articles were as follows (Cui et al., 2014): the studies must be RCTs (Ahmed and Al-Sayed, 2010); each article provided authentic and valid data (Raheem et al., 2017); participants were patients diagnosed with ureteral stones and met the MET criteria; and (Thongprayoon et al., 2020) the studies compared mirabegron treatments with α-adrenergic antagonist in patients with ureteral stones. Consequently, we excluded any clinical studies where non-α-adrenergic antagonist were used in the control group. In cases where the same research was published in multiple journals or at different times, the most recent version was chosen for the meta-analysis. Additionally, case reports, review articles, meeting abstracts, conference reports and studies lacking sufficient data were excluded. The details of the inclusion and exclusion criteria are shown in Supplementary Table 2.

### Quality assessment

Two authors independently evaluated the risk of bias in this study using the Cochrane Risk of Bias (RoB) 2.0 tool, focusing on multiple domains: the randomization process, deviations from the intended interventions, incomplete outcome data, outcome measurement, and selection of reported results (Higgins et al., 2011). Any disagreements between the reviewers were resolved through discussions with a third investigator. Each domain received a rating of “low,” “some concerns,” or “high” risk. The overall bias risk for each study was determined based on the highest risk level assigned in any domain.

### Data extraction and outcome measures

Two authors independently extracted data from the included articles, organizing information such as the author’s name, publication year, country, sample size, treatments and comparators, study duration, stone location, and stone size. The primary outcome measured was the stone expulsion rate (SER), while secondary outcomes included the stone expulsion interval (SEI) and the number of pain episodes during follow-up. If a study did not report standard deviations (SD), these were derived from the provided standard errors (SE), confidence intervals (CI), or P values. In cases where none of these values were available, the SD was estimated using correlation coefficients from similar studies.

### Statistical analyses

Data analysis for this study was conducted using Review Manager version 5.3.0 (Cochrane Collaboration). For dichotomous outcomes, the risk ratio (RR) was calculated, while the mean difference (MD) was used for continuous outcomes, both reported with 95% confidence intervals (CIs). To assess statistical heterogeneity, Cochran’s Q test and the I^2^ statistic were applied, with heterogeneity defined as I^2^ > 50% or p < 0.05. When no heterogeneity was detected, a fixed-effects model was used to combine the effect sizes; otherwise, a random-effects model was applied. P < 0.05 was considered statistically significant.

## Results

### Characteristics of included studies

**FIGURE 1:**
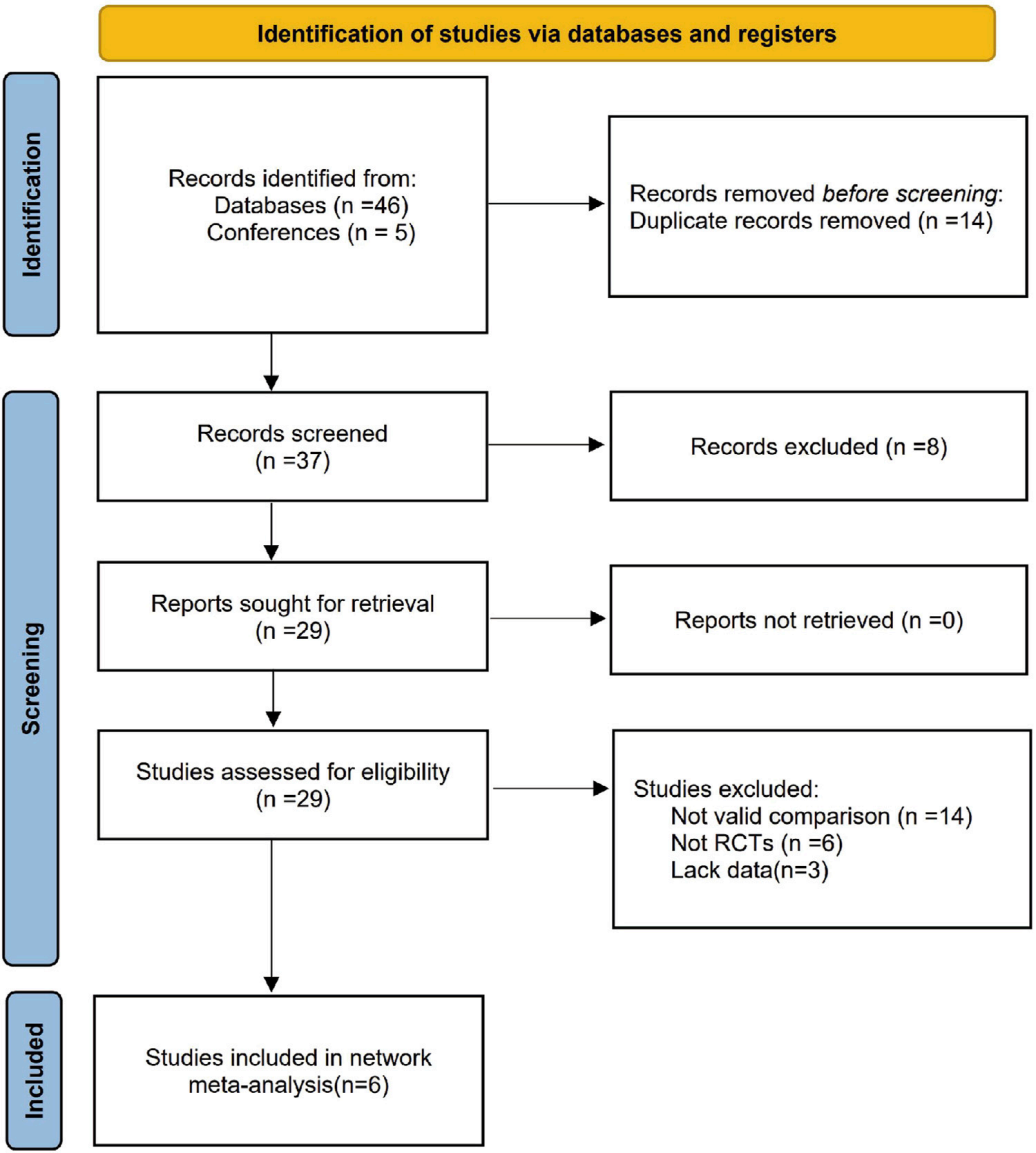
PRISMA flow diagram.

We initially identified 51 articles through our search strategy, but 22 were removed after screening their titles and abstracts. Out of the 29 articles left, 20 were excluded for not meeting the inclusion criteria, and an additional 3 from the remaining 9 were eliminated due to insufficient data. Ultimately, 6 studies were included in our analysis to assess the efficacy of mirabegron and α-adrenergic receptor antagonist in treating distal ureteral stones (Abdel-Kader et al., 2024; Bayar et al., 2020; Faridi and Deshpande, 2024; Morsy et al., 2022; Samir et al., 2024; Seleem et al., 2021). The study selection process is illustrated in Figure 1, with detailed characteristics of these studies provided in Table 1.

**TABLE 1:** Characteristics of included studies.

Study (years)	Country	Study design	Sample size		Intervention		Duration	Stone size	Stone location
			Trial	Control	Trial	Control			
Bayar et al. (2020)	Turkey	RCT	29	35	Mirabegron (50 mg/day)	Silodosin (8 mg/day)	4 weeks	4–10 mm	Distal ureter
Faridi and Deshpande (2024)	India	RCT	56	58	Mirabegron (50 mg/day)	Silodosin (8 mg/day)	4 weeks	5–10 mm	Distal ureter
Abdel-Kader et al. (2023)	Egypt	RCT	35	35	Mirabegron (50 mg/day)	Silodosin (8 mg/day)	4 weeks	≤10 mm	Distal ureter
Samir et al. (2024)	Egypt	RCT	57	59	Mirabegron (50 mg/day)	Silodosin (8 mg/day)	4 weeks	5–10 mm	Distal ureter
Morsy et al. (2022)	Egypt	RCT	25	25	Mirabegron (50 mg/day)	Tamsulosin (0.4 mg/day)	30 days	<10 mm	Distal ureter
Seleem et al. (2021)	Egypt	RCT	37	36	Mirabegron (50 mg/day)	Tamsulosin (0.4 mg/day)	NA	5–10 mm	Distal ureter

### Risk of bias

**FIGURE 2:**
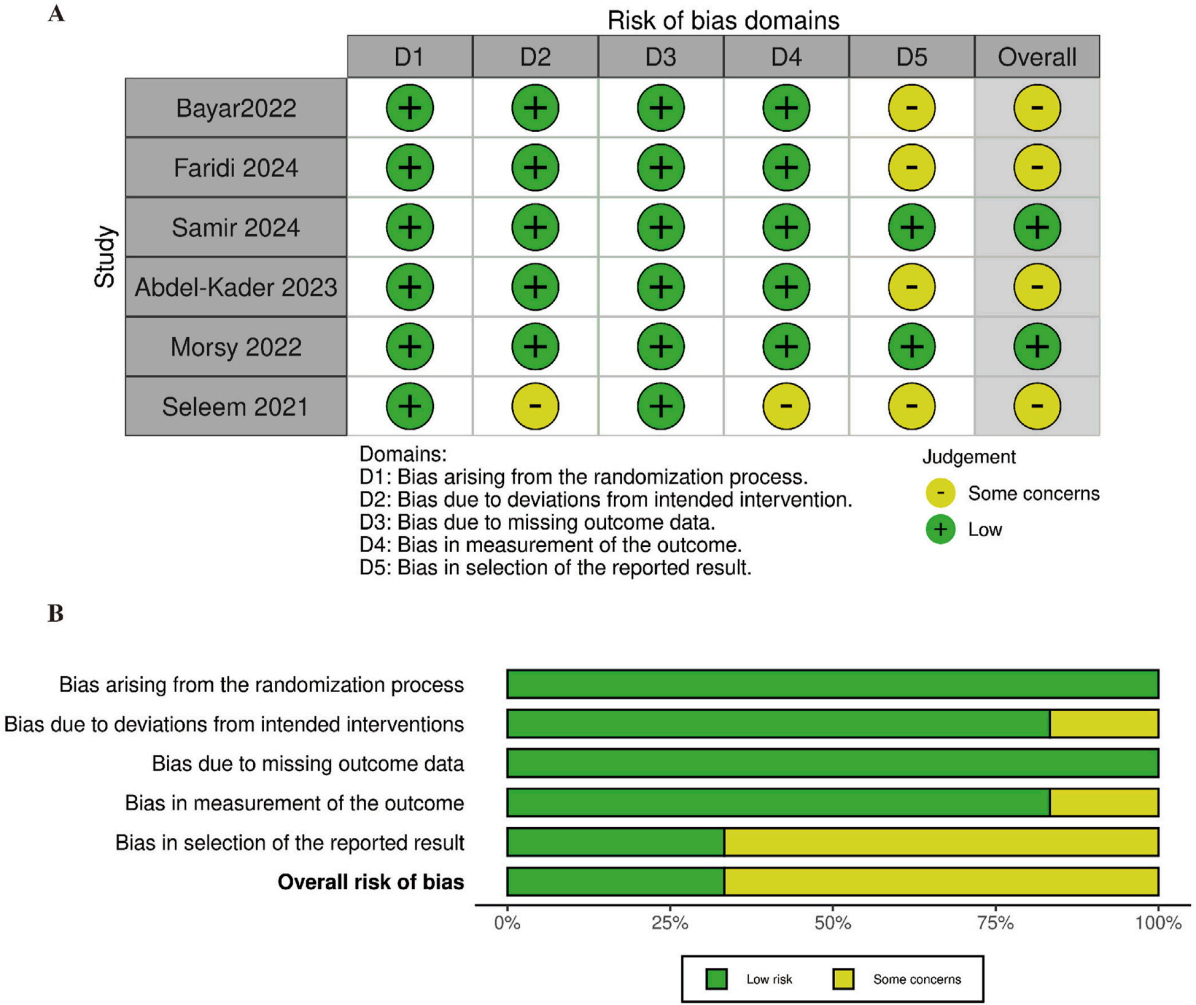
The assessment of risk of bias (RoB). **(A)** Risk of bias domain for each included study; **(B)** Summary of risk of bias assessment.

**FIGURE 3:**
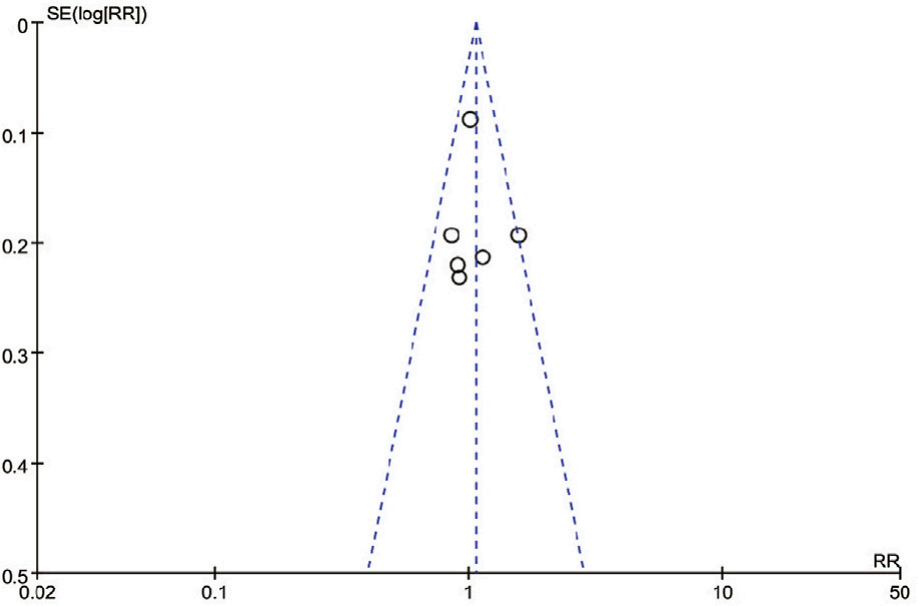
Funnel plot of the articles.

The risk of bias (RoB) assessment for each study is shown in Figure 2. Out of the included studies, 4 were found to have some concerns, while the remaining 2 were considered to have a low RoB. The most common sources of potential bias were related to the randomization process and the selection of reported outcomes. The bias analysis produced highly symmetrical plots, consisting of 6 squares representing studies that evaluated the efficacy of mirabegron and α-adrenergic receptor antagonist in treating distal ureteral stones (Figure 3).

### Assessment of efficacy

#### SER

**FIGURE 4:**
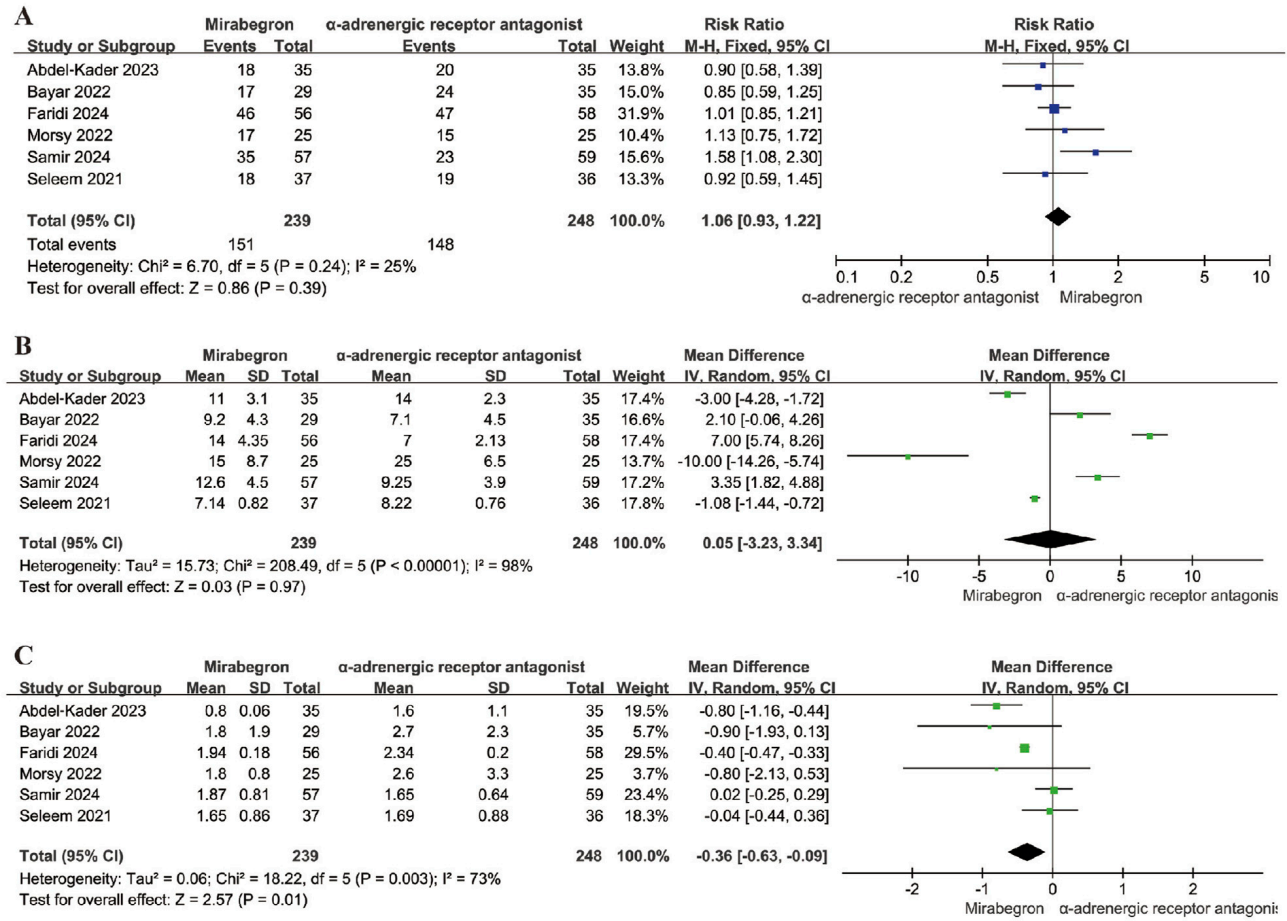
Forest plots showing the pooled results of SER, SEI and pain episodes between mirabegron and α-adrenergic receptor antagonist group. **(A)** SER; **(B)** SEI; **(C)** pain episodes. SER, stone expulsion rate; SEI, stone expulsion interval.

Six studies, involving 487 participants (239 in the mirabegron group and 248 in the α-adrenergic receptor antagonist treatment group), provided data on the SER when comparing mirabegron to α-adrenergic receptor antagonist. A fixed effects model was applied to calculate the RR with a 95% CI, considering no heterogeneity (p = 0.24; I^2^ = 25%). The analysis showed that mirabegron did not significantly increase the SER compared to the α-adrenergic receptor antagonist group (RR = 1.06; 95% CI = 0.93–1.22; P = 0.39) (Figure 4A). Sensitivity analysis, in which each study was sequentially excluded and the pooled RR recalculated, consistently supported the original findings (Supplementary Figure S1A), confirming the stability of the meta-analysis results for SER outcomes.

### SEI

Six studies, including 487 participants (239 in the mirabegron group and 248 in the α-adrenergic receptor antagonist group), presented data on the SEI in a comparison between mirabegron and α-adrenergic receptor antagonist. A random effects model was utilized to calculate the MD with a 95% CI, taking into account substantial heterogeneity (Q = 208.49; p < 0.0001; I^2^ = 98%). The results indicated that mirabegron did not significantly shorten the SEI compared to the α-adrenergic receptor antagonist group (MD = 0.05; 95% CI = −3.23 to 3.34; P = 0.97) (Figure 4B). Sensitivity analysis, which involved recalculating the pooled MD after excluding each study one at a time, consistently supported the initial findings (Supplementary Figure S1B), reinforcing the reliability of the meta-analysis results regarding SEI.

#### Frequency of pain episodes

Six studies, involving 487 participants (239 in the mirabegron group and 248 in the α-adrenergic receptor antagonist group), provided data on the frequency of pain events during MET when comparing mirabegron to α-adrenergic receptor antagonist. A random effects model was applied to calculate the MD with a 95% CI, accounting for moderate heterogeneity (Q = 18.22; p = 0.003; I^2^ = 73%). The analysis revealed that mirabegron significantly reduced the frequency of pain episodes compared to the α-adrenergic receptor antagonist group (MD = −0.36; 95% CI = −0.63 to −0.09; P = 0.01) (Figure 4C). Sensitivity analysis, in which each study was sequentially excluded and the pooled MD recalculated, consistently confirmed the original findings (Supplementary Figure S1C), highlighting the robustness of the meta-analysis results regarding pain episode frequency.

### Assessment of safety

#### Headache

**FIGURE 5:**
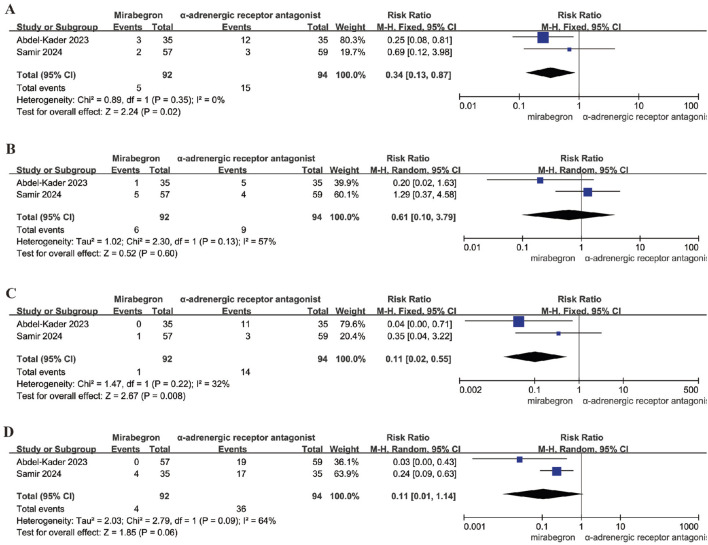
Forest plots showing the pooled results of headache, dizziness, orthostatic hypotension, ejaculation dysfunction. **(A)** headache; **(B)** dizziness; **(C)** orthostatic hypotension; **(D)** ejaculation dysfunction.

Two studies, involving 186 participants (92 in the mirabegron group and 94 in the α-adrenergic receptor antagonist group), provided data on the incidence of headache. A fixed effects model was utilized to calculate the RR with a 95% confidence interval CI (p = 0.35; I^2^ = 0). The analysis revealed a significant difference between the mirabegron and α-adrenergic receptor antagonist groups (RR = 0.34; 95% CI = 0.13–0.87; P < 0.05) (Figure 5A). This indicates that mirabegron is associated with a lower frequency of headache compared to the α-adrenergic receptor antagonists.

#### Dizziness

Two studies, with a total of 186 participants (92 in the mirabegron group and 94 in the α-adrenergic receptor antagonist group), reported on the occurrence of dizziness. A random effects model was utilized to calculate the RR with a 95% CI (Q = 2.3; p = 0.13; I^2^ = 57%). The results indicated no significant difference between the mirabegron and α-adrenergic receptor antagonist groups (P = 0.60) (Figure 5B). This implies that the rates of dizziness are comparable between both treatment options.

#### Orthostatic hypotension

Two studies examined orthostatic hypotension, involving 186 participants (92 in the mirabegron group and 94 in the α-adrenergic receptor antagonist group). A fixed effects model was applied to compute the RR with a 95% CI (p = 0.22; I^2^ = 32%). The analysis revealed a significant difference between the mirabegron and α-adrenergic receptor antagonist groups (RR = 0.11; 95% CI = 0.02–0.55; P = 0.008) (Figure 5C). This indicates that mirabegron is associated with a lower incidence of orthostatic hypotension compared to the α-adrenergic receptor antagonists.

#### Ejaculation dysfunction

Two studies also focused on ejaculation dysfunction, involving a total of 186 participants (92 in the mirabegron group and 94 in the α-adrenergic receptor antagonist group). A random effects model was used to calculate the RR with a 95% CI (Q = 2.79; p = 0.09; I^2^ = 64%). The findings indicated no significant difference between the mirabegron and α-adrenergic receptor antagonist groups (P = 0.06) (Figure 5D). This suggests that the prevalence of ejaculation dysfunction is similar for both treatment options.

### Subgroup analysis

This study utilized 2 types of α-adrenergic blockers—tamsulosin and silodosin—for the treatment of distal ureteral stones. A subgroup analysis was conducted to account for the differences between these medications.

### SER

**FIGURE 6:**
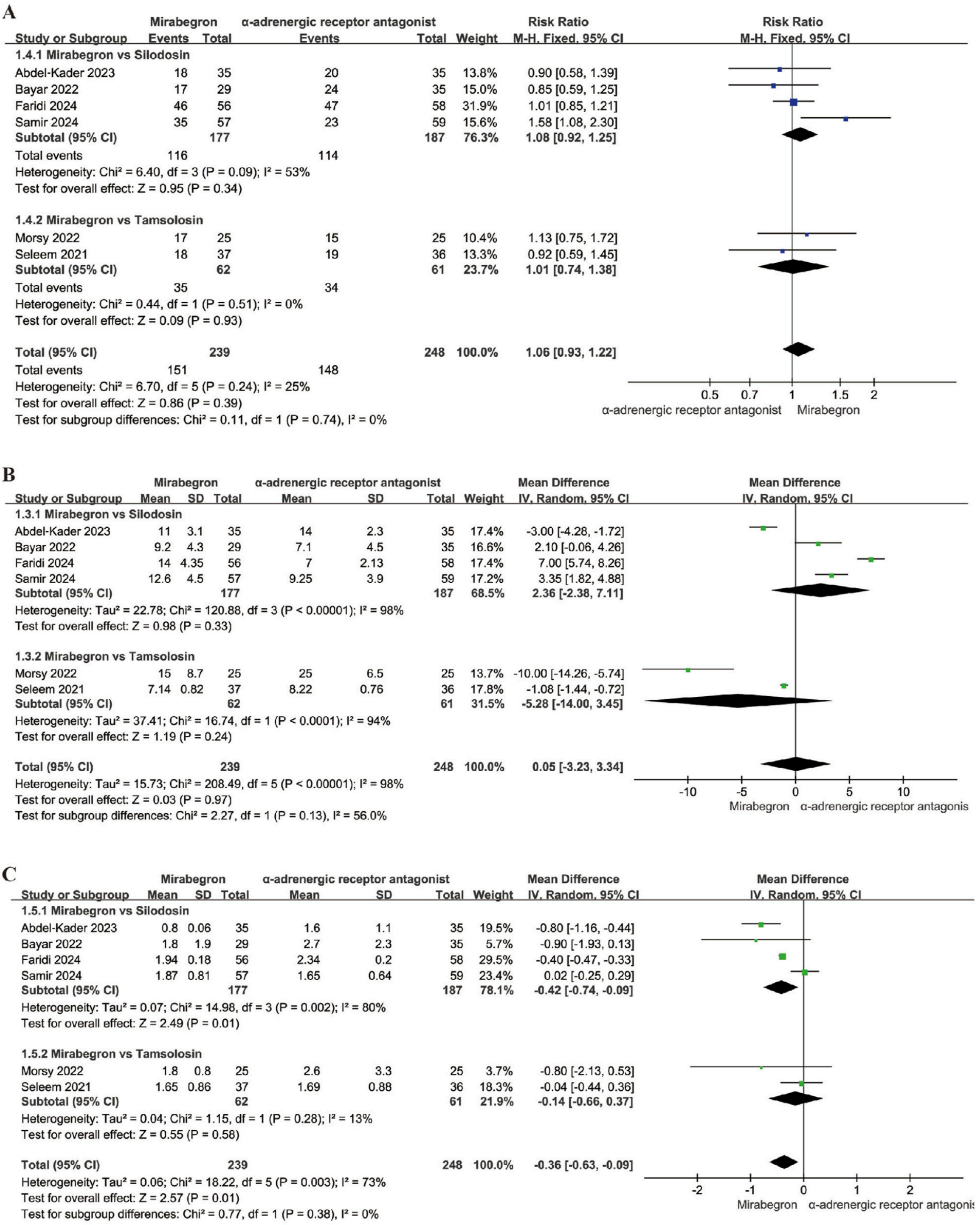
Forest plots showing the subgroup analysis based on types of α-adrenergic receptor antagonist. **(A)** SER; **(B)** SEI; **(C)** pain episodes. SER, stone expulsion rate; SEI, stone expulsion interval.

In this evaluation, 4 RCTs comparing mirabegron with silodosin and 2 RCTs comparing mirabegron with tamsulosin were included. We found no significant difference between mirabegron and silodosin in SER (RR = 1.08; 95% CI = 0.92–1.25; P = 0.34). The comparison with tamsulosin showed similar results (RR = 1.01; 95% CI = 0.74–1.38; P = 0.93) (Figure 6A).

### SEI

This assessment incorporated 4 RCTs comparing mirabegron with silodosin and 2 RCTs comparing mirabegron with tamsulosin. The analysis revealed no significant difference in the SEI between mirabegron and silodosin (MD = 2.36; 95% CI = −2.38 -7.11; P = 0.33). Similarly, the comparison with tamsulosin yielded comparable results (MD = −5.28; 95% CI = -14 - 3.45; P = 0.24) (Figure 6B).

#### Frequency of pain episodes

In this analysis, 4 RCTs comparing mirabegron with silodosin and 2 RCTs comparing mirabegron with tamsulosin were included. We observed a significant difference in the frequency of pain events during medical MET between mirabegron and silodosin (MD = −0.42; 95% CI = −0.74 to −0.09; P = 0.01). In contrast, the comparison with tamsulosin did not reveal any significant difference in the frequency of pain episodes (MD = −0.14; 95% CI = −0.66-0.37; P = 0.58) (Figure 6C).

## Discussion

Current clinical guidelines indicate that MET is a viable treatment option for distal ureteral stones (Beach and Mauro, 2006). α-adrenergic receptor blockers, such as tamsulosin, are commonly utilized in MET. Additionally, other medications, including calcium channel blockers, phosphodiesterase type 5 inhibitors, and corticosteroids, have also demonstrated efficacy in facilitating the passage of ureteral stones (Itoh et al., 2011). Recently, studies have explored the potential of mirabegron, a β3-adrenergic receptor agonist, in promoting stone expulsion (Shen et al., 2017). A meta-analysis by Song et al. concluded that mirabegron significantly increased the SER of distal ureteral stones and reduced the frequency of pain episodes (Song et al., 2023).However, the effectiveness of mirabegron remains a subject of debate. While some randomized controlled trials have reported benefits of mirabegron in stone expulsion, others have not confirmed these findings (Van Asseldonk and Elterman, 2015; Ye et al., 2011). For instance, Tang et al. demonstrated that mirabegron significantly improved SER in patients with stones measuring ≤5 mm but had no effect on those with larger stones (Tang et al., 2021). In this meta-analysis, we systematically evaluated the efficacy and safety of mirabegron compared to α-adrenergic receptor blockers for the treatment of distal ureteral stones.

Our findings indicated no significant differences in SER or SEI between the mirabegron and α-adrenergic receptor antagonist groups. This suggests that mirabegron may have comparable overall efficacy to tamsulosin and silodosin regarding stone passage and expulsion time. Notably, our analysis revealed that mirabegron significantly reduced the frequency of pain episodes during stone expulsion, particularly in comparison to silodosin. Although pain perception is a subjective outcome measure—unlike objective metrics such as SER or SEI—it remains a critical endpoint in urolithiasis management. Patient-reported pain relief directly reflects therapeutic success from the patient’s perspective, as uncontrolled colic not only diminishes quality of life but also drives healthcare resource utilization (Cabo and Miller, 2024). This reduction in pain frequency holds direct clinical relevance: fewer pain episodes may translate into improved quality of life by reducing dependence on analgesics (e.g., opioids or NSAIDs), decreasing emergency department visits for uncontrolled colic, and enabling earlier resumption of daily activities or occupational duties. The result underscores the potential advantage of mirabegron in pain management during MET.

The use of β3-adrenergic receptor agonists, such as mirabegron, is primarily associated with the management of overactive bladder (Lipkin and Shah, 2009). However, recent research has identified the presence of β-adrenergic receptors in the smooth muscle of the human ureter (Taylor et al., 2004). The activation of β3-adrenergic receptors is believed to relax ureteral smooth muscle, facilitating stone passage by reducing the frequency of peristaltic contractions (Tomiyama et al., 2007). This mechanism may account for the observed reduction in pain frequency in our analysis, as fewer ureteral contractions can lead to less discomfort during stone expulsion.

In terms of adverse effects, the analysis revealed that mirabegron had a significantly lower incidence of headache and orthostatic hypotension compared to α-adrenergic receptor antagonists, suggesting that mirabegron may have a more favorable safety profile. However, there were no significant differences between the two groups in terms of dizziness or ejaculation dysfunction, further supporting the potential suitability of mirabegron for certain patients. It is important to note that the safety assessment was based on only two included studies. While meta-analyses can be conducted with as few as two studies, the low heterogeneity observed here should be interpreted cautiously. A low I^2^ value does not necessarily indicate the absence of true heterogeneity; it may instead reflect limited statistical power due to the small number of trials or insufficient variability in study designs. The reduced side effects of mirabegron, particularly in relation to headache and hypotension, are likely due to its targeted action on β3 receptors in the bladder, with minimal influence on the vascular system (Shen et al., 2017). In contrast, α1-adrenergic receptor antagonists act by blocking α1 receptors in vascular smooth muscle, leading to vasodilation and lower blood pressure, which increases the risk of orthostatic hypotension and headache (Itoh et al., 2011; Tomiyama et al., 2007). Overall, both mirabegron and α-adrenergic receptor antagonists were generally well tolerated by patients, with mild side effects commonly reported. Nevertheless, the limited scope of safety data underscores the need for future trials to prioritize standardized reporting of adverse events across larger cohorts, which would enhance the reliability of safety comparisons. This suggests that mirabegron may serve as an alternative treatment option for patients with contraindications to α-adrenergic receptor antagonists or for those who do not respond well to initial therapy.

In our subgroup analysis, we compared mirabegron with both tamsulosin and silodosin. Interestingly, while mirabegron showed a significant improvement in pain relief compared to silodosin, no significant differences were noted between mirabegron and tamsulosin across any of the studied parameters, including SER, SEI, or pain episodes. This suggests that the therapeutic effects of mirabegron may be more comparable to tamsulosin but could offer superior pain relief compared to silodosin. However, further studies are required to confirm these findings and explore the underlying mechanisms.

Our study has several limitations. Firstly, the small number of studies and limited sample size reduce the statistical power of the analysis. Secondly, the high heterogeneity in some endpoints, potentially due to variations in study design, sample size, and inclusion criteria, weakens the overall reliability of our findings. Additionally, most of the included studies were conducted in specific regions, limiting the generalizability of the results to broader populations.

## Conclusion

Mirabegron may offer advantages in managing pain during medical expulsive therapy for distal ureteral stones, especially when compared to silodosin, even though no significant differences were observed in SER or SEI. Furthermore, mirabegron demonstrated a favorable safety profile, showing reduced rates of headache and orthostatic hypotension relative to α-adrenergic receptor antagonists. To validate these results and explore the mechanisms behind the different impacts on pain relief and safety, additional well-structured randomized controlled trials are necessary.

## Data Availability

The original contributions presented in the study are included in the article/Supplementary Material, further inquiries can be directed to the corresponding authors.
