# Supplementary figures and images for: The efficacy and safety of mirabegron and α-adrenergic receptor antagonist in the treatment of distal ureteral stones: a systematic review and meta-analysis
Source: Front Pharmacol. 2025 Mar 19;16:1517979. doi: 10.3389/fphar.2025.1517979 (PMC11961960; doi:10.3389/fphar.2025.1517979)

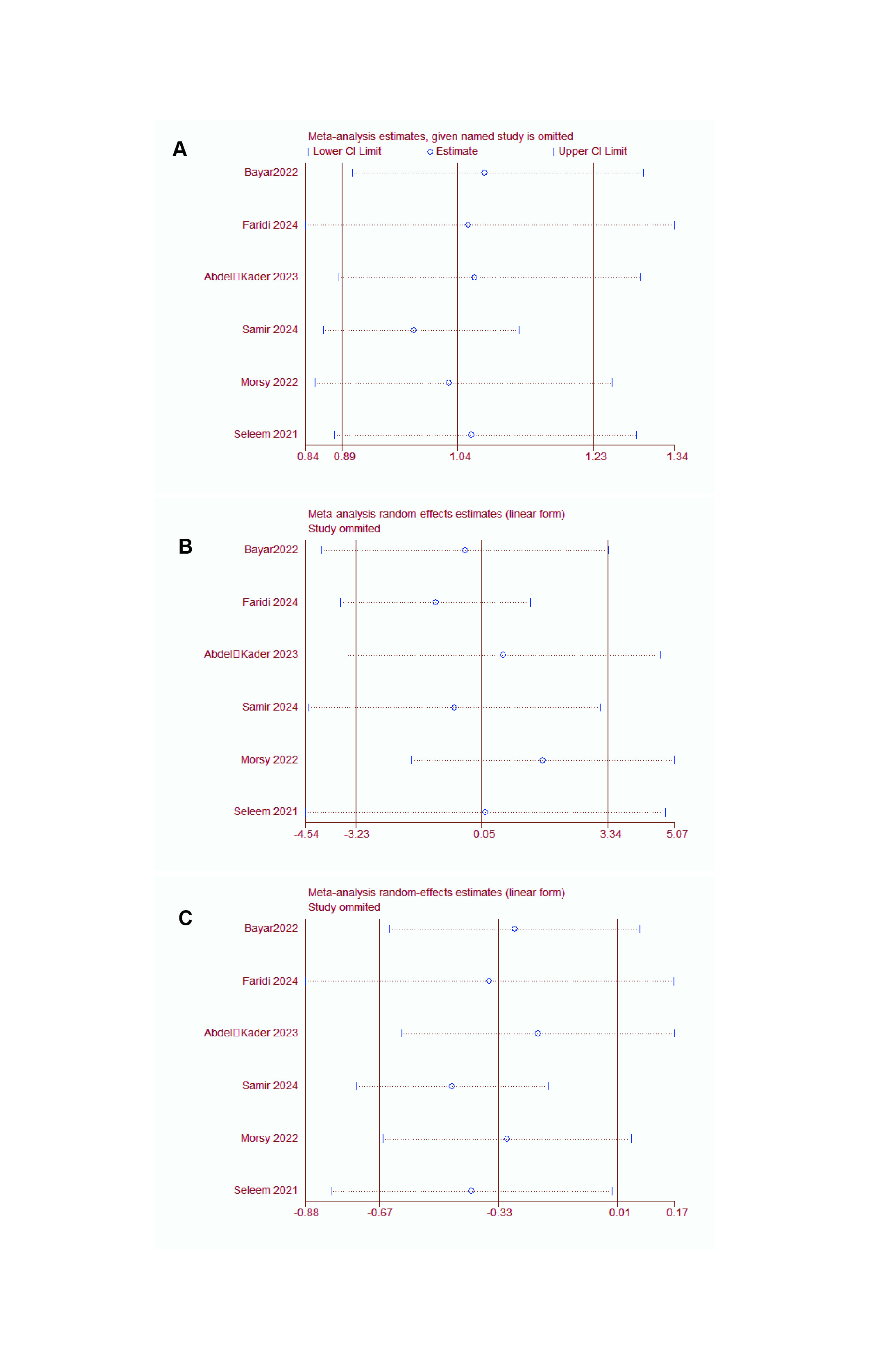

Supplement: Supplementary file 3 [file Image1.jpeg]
