# Supplementary material for: The efficacy and safety of mirabegron and α-adrenergic receptor antagonist in the treatment of distal ureteral stones: a systematic review and meta-analysis
Source: Front Pharmacol. 2025 Mar 19;16:1517979. doi: 10.3389/fphar.2025.1517979 (PMC11961960; doi:10.3389/fphar.2025.1517979)
Supplement: Supplementary file 4 [file Table2.docx]

**Supplementary Table 2: Search strategy according to populations, interventions, comparators, outcomes, and study designs (PICOS)**

|  | Population | Intervention | Comparator | Outcomes | Study Design |
| --- | --- | --- | --- | --- | --- |
| Inclusion Criteria | Patients, aged more than 18-year-old, presented with a lower ureteric stone less than 10 mm in size, diagnosed by ultrasound (USG) abdomen/pelvis or kidney-ureter-bladder (KUB) X-ray, or computed tomography (CT) scan are included. | mirabegron | α-adrenergic antagonist | The main effects (including expulsion rate, expulsion time, frequency of pain episodes) and side effects (including headache, dizziness, abnormal ejaculation, orthostatic hypotension) | Prospective randomized clinical research |
| Exclusion Criteria | Patients who were diagnosed with nephrolithiasis, > 10 mm ureteral stones, bilateral ureteral stones, ureteral stones requiring urgent intervention or obstructive, hydronephrosis, multiple ureteral stones and any anatomical abnormalities on CT examination are excluded.  Patients with UTI, deteriorating renal function, fever, acute or chronic renal failure, a history of open surgery or endoscopic procedures in the urinary tract, allergy to tamsulosin or tadalafil, concomitant treatment with calcium antagonists, or nitrates, pregnant or lactating mothers are also excluded. | Not performed | non-α-adrenergic antagonist | Qualitative outcomes such as patient feelings; inadequate indicators; | Letters, comments, reviews, qualitative studies |
